# Short-term sensorimotor practice reduces somatosensory temporal discrimination threshold and enhances somatosensory cortex excitability

**DOI:** 10.1016/j.cnp.2026.04.008

**Published:** 2026-04-28

**Authors:** Naoshin Yoshida, Tomotaka Suzuki, Kakuya Ogahara, Tomoya Kokue, Toshio Higashi, Kenichi Sugawara

**Affiliations:** a Graduate School of Biomedical Sciences, Nagasaki University, 1-7-1 Sakamoto, Nagasaki, Nagasaki Prefecture, Japan; b Department of Rehabilitation, Yokosuka Kyosai Hospital, 1-16 Yonegahamadori, Yokosuka, Kanagawa Prefecture, Japan; c Graduate Course of Health and Social Services, Kanagawa University of Human Services, 1-10-1 Heisei-cho, Yokosuka, Kanagawa Prefecture, Japan

**Keywords:** Somatosensory temporal discrimination threshold, Sensorimotor practice, Primary somatosensory cortex, Paired-pulse depression, High-frequency oscillations, Intracortical inhibition

## Abstract

**Objective:**

To investigate whether short-term sensorimotor practice alters the somatosensory temporal discrimination threshold (STDT), primary somatosensory cortex (S1) responses, and intracortical inhibition.

**Methods:**

Thirty-eight healthy adults were randomly assigned to either a coin rotation (CR, n = 19) or a finger tapping (FT, n = 19) group and completed 10 training sessions. CR performance, STDT, somatosensory evoked potentials (SEPs), high-frequency oscillations (HFOs), and paired-pulse depression (PPD) at interstimulus intervals (ISI) of 5 ms and 30 ms were assessed before and after training.

**Results:**

CR performance improved by 33% (*p* < 0.001). STDT decreased on the trained side (main effect of test: *p* = 0.017), with no between-group difference or interaction. N20/P25 amplitude and HFOs (single-stimulus and ISI 5 ms conditions) increased after training. PPD at both ISIs showed no significant effects.

**Conclusions:**

Short-term sensorimotor practice reduced STDT and enhanced S1 responses without detectable changes in PPD-indexed inhibition. The lack of between-group differences suggests these effects may reflect a common effect of repetitive sensorimotor practice across the two active tasks tested, rather than task-specific learning.

**Significance:**

In this paradigm, STDT improved without detectable changes in PPD-indexed inhibition, suggesting that alternative mechanisms of sensory temporal processing warrant consideration.

## Highlights

• Short-term practice reduced somatosensory temporal discrimination threshold.
• Somatosensory cortex excitability and high-frequency oscillations increased.
• Paired-pulse depression reflecting intracortical inhibition was unchanged.

## 1 Introduction

Sensory information plays an essential role in motor control and learning by supporting movement correction and monitoring. The somatosensory temporal discrimination threshold (STDT) quantifies temporal tactile processing capacity by identifying the minimum time interval at which two consecutively presented stimuli are perceived as separate events. STDT is thought to reflect inhibitory interneuron activity in the primary somatosensory cortex (S1) and is modulated by an extensive basal ganglia-thalamus-cortex network (Lee et al., 2018, Ordás and Alonso-Frech, 2024). A close relationship exists between motor execution and the STDT. In healthy individuals, STDT values transiently increase during movement (0–200 ms post-onset), reflecting sensory gating associated with motor execution, which may be related to surround inhibition in the motor system and contribute to efficient motor performance (Conte et al., 2016, Belvisi et al., 2018, Manzo et al., 2023). In patients with Parkinson's disease, STDT correlates with performance on the coin rotation (CR) task, which requires feedback control, and with variability in finger tapping (FT), a task that predominantly relies on feedforward control (Lee et al., 2010, Lee et al., 2016). These findings indicate that temporal processing of tactile information contributes to sensorimotor integration in dexterity tasks. Furthermore, considerable reductions in STDT values following motor learning in feedback-controlled tasks have been previously reported (Yoshida et al., 2020, Zhang et al., 2025).

A previous study also suggests that S1 excitability and inhibition-related activity may change during motor learning (Pham et al., 2022). In addition, changes in STDT have been observed following theta burst stimulation (TBS) of S1 (Conte et al., 2012, Conte et al., 2014, Conte et al., 2016, Rocchi et al., 2016) and after high-frequency cutaneous stimulation (Erro et al., 2016, Rocchi et al., 2017). However, the central nervous mechanisms underlying STDT regulation during motor learning, including changes in S1 intracortical inhibition, remain poorly understood.

Based on previous findings that (1) STDT correlates with dexterity in feedback-controlled tasks (Lee et al., 2010, Lee et al., 2016), (2) S1 intracortical inhibition modulates STDT (Lee et al., 2018, Ordás and Alonso-Frech, 2024), and (3) motor learning induces S1 plasticity (Pham et al., 2022), we hypothesized that CR training, given its greater reliance on online sensory feedback, might induce larger changes in STDT and PPD-indexed S1 inhibitory processing than FT. We also expected the CR group to show a greater magnitude of change overall. Nevertheless, because FT is a rhythmic sensorimotor task that engages sensorimotor networks partially overlapping with those recruited during CR, between-group differences may be constrained.

This study aimed to examine the changes in S1 intracortical inhibition, cortical excitability, and STDT associated with sensorimotor practice.

## 2 Methods

### 2.1 Participants

Thirty-eight healthy adults with no history of neurological or psychiatric disorder participated in this study. Participants were randomly assigned to either the coin rotation (CR) group (n = 19; age: 22.9 ± 3.2 years; 17 males, 2 females; 18 right-handed, 1 left-handed) or the finger tapping (FT) group (n = 19; age: 22.4 ± 5.1 years, 13 males, 6 females; 17 right-handed, 2 left-handed). Handedness was assessed using the Oldfield Handedness Inventory (Oldfield, 1971). All participants provided written informed consent. The study was approved by the Ethics Committee of Kanagawa University of Human Services (approval number: 5-21-29) and conducted in accordance with the Declaration of Helsinki.

### 2.2 Protocol

Each participant completed 10 sessions, each consisting of 2 min of training followed by 1 min of rest. The CR test, STDT, and somatosensory evoked potential (SEP) measurements were performed before (pre-test) and after (post-test) the training period.

### 2.3 Training tasks

#### 2.3.1 CR group

Participants in the CR group performed the CR task (Mendoza et al., 2009) using their non-dominant hand. Using the thumb, index, and middle fingers, they repeatedly rotated a 5-cent coin by 180°, either toward or away from the body. The rotation direction remained consistent throughout training and testing. The CR task commenced immediately after the pre-test and consisted of 10 2-min sessions with 1-min rest intervals. Training was conducted without visual feedback of hand position. Participants were informed of their pre-test results and instructed to improve performance during training. They were also instructed not to use their non-training hand during the training or rest periods.

#### 2.3.2 FT group

The FT group performed the FT task. Participants tapped a fixed 5-cent coin with their thumb and index finger and thumb and middle finger at approximately 1 Hz without synchronizing to external cues. Ten sessions of 2-min tapping with 1-min rest periods were performed. FT was selected as an active comparison task to match motor activity and training duration while imposing lower intended feedback demands than CR, although substantial overlap with CR-related sensorimotor networks was anticipated.

### 2.4 CR test

The CR test has been used in previous studies to assess the relationship between STDT and finger dexterity (Lee et al., 2010, Lee et al., 2016). Participants performed 180° CRs as quickly as possible for 10 s (Gebhardt et al., 2008, Lee et al., 2010, Lee et al., 2016), and the number of rotations was counted. After a 10-s rest, the test was repeated three consecutive times. If the coin was dropped onto the table, participants retrieved it immediately without stopping the timer. Start and stop cues were provided audibly. The test was performed without visual feedback. The CR score was calculated as follows: CR score = half turns − ([coin drops × 0.1] × half turns) (Gebhardt et al., 2008). The average of the three CR scores was used for analysis.

### 2.5 STDT procedure

STDT measurements were performed on both index fingers. Paired tactile electrical stimuli (duration, 0.2 ms) were delivered through 1-mm-diameter electrodes (Unique Medical Co., Kyushu, Japan) placed 5 mm apart on the distal volar surface of each finger using a pulse generator and isolator (Nihon Kohden Co., Tokyo, Japan).

STDT was determined using the method of limits. Stimulus intensity was increased in 1-mA increments from 0 mA until the minimum intensity detected 10 out of 10 times was established; stimulation was then applied at approximately 1.5 times this threshold. The same stimulus intensity was used for pre- and post-tests. Interstimulus interval (ISI) increased from 0 ms in 5-ms increments. The first ISI at which two consecutive stimuli were recognized as separate on three consecutive trials was defined as the ascending temporal discrimination threshold (ATDT) (Rocchi et al., 2016). Next, ATDT + 100 ms was decreased to determine the descending temporal discrimination threshold. Each procedure was repeated three times, and STDT was calculated as the average of all six threshold values (Lacruz et al., 1991, Yoshida et al., 2020).

To maintain attention and reduce perseverative responses (Conte et al., 2016), catch trials with single stimuli were presented at a frequency of 20% using a pulse generation system (LabVIEW 2009, National Instruments, Austin, TX, USA).

### 2.6 SEP procedure

SEP recordings were conducted following procedures used in previous STDT studies (Paparella et al., 2020, Rocchi et al., 2024). Silver chloride disc electrodes were fixed with conductive paste according to the international 10–20 system: reference electrode at Fpz and active electrode at C4′ or C3′ (2 cm posterior to C4 or C3). SEP, HFO, and PPD recordings were obtained from the hemisphere contralateral to the trained hand, with median nerve stimulation delivered to the trained side; this procedure was applied uniformly regardless of handedness. Electrode–skin impedance was maintained below 5 kΩ. Stimulation electrodes were fixed with Velcro bands over the median nerve at the wrist. Stimulus intensity produced a visible abductor pollicis brevis contraction without pain, and the same stimulus intensity was used for pre- and post-tests. Stimulus duration was 0.2-ms rectangular pulses. A total of 1,500 stimuli were administered: 500 single stimuli and 500 paired stimuli at ISIs of 5 ms (ISI5ms) and 30 ms (ISI30ms), delivered randomly. The stimulation rate was 3 Hz. SEP waveforms were sampled at 4 kHz with a 5–2000-Hz band-pass filter, and 500 responses were averaged for each condition. The recording window spanned from 20 ms before to 100 ms after stimulation.

Inhibition at short ISIs is thought to primarily reflect local inhibitory mechanisms within S1, whereas inhibition at long ISIs is considered to reflect more complex inhibitory loops involving remote structures outside S1, such as the dorsal column nuclei (Lüders et al., 1984) and the thalamus (Höffken et al., 2010, Emori et al., 1991, Ugawa et al., 1996, Mochizuki et al., 2001, Rocchi et al., 2024).

ISI5ms has been reported to correlate with the STDT in previous studies (Rocchi et al., 2016, Rocchi et al., 2017, Yamazaki et al., 2020) and has been used as an index of local inhibition within S1. Furthermore, it has been demonstrated that inhibition at ISI5ms and ISI30ms is modulated by differences in aerobic exercise intensity (Yamazaki et al., 2020) and high-frequency repetitive somatosensory stimulation (HF-RSS) (Rocchi et al., 2017).

Based on the above, and following Paparella et al. (2020) and Rocchi et al. (2024), ISI5ms was selected as an index of local inhibitory processing within S1, and ISI30ms was selected as an index of more complex inhibitory loops involving remote structures outside S1. However, it should be noted that although Paparella et al. (2020) and Rocchi et al. (2024) assumed that inhibition at 20–40 ms reflects mechanisms similar to inhibitory loops outside S1 and employed ISI30ms in their experiments based on this assumption, the underlying mechanism of inhibition at ISI30ms has not been directly demonstrated.

The N20/P25 peak-to-peak amplitude was used as an index of sensory cortex excitability.

For PPD analysis, the single-stimulus SEP waveform was subtracted from the paired-stimulus waveform. S1 intracortical inhibition was quantified as the suppression ratio of the second response in the subtracted paired-stimulus SEP waveform relative to the single stimulus. Indices derived from SEP ISI5ms and ISI30ms conditions were designated PPD5ms and PPD30ms, respectively.

For high-frequency oscillation (HFO) analysis, the stimulus artifact was removed manually from −10 to +5 ms to avoid ringing artifacts (Katayama et al., 2010). The broadband SEP signals were band-pass filtered at 400–800 Hz using a zero-phase, bidirectional Butterworth filter and subsequently averaged. The N20 peak latency and HFOs were then identified from the averaged waveforms.

The HFO waveform was separated into two components based on the N20 peak latency: an early high-frequency oscillation (eHFO) and a late high-frequency oscillation (lHFO). The onset of the eHFO and the offset of the lHFO were defined as the time points at which the amplitude exceeded the mean background noise level by more than 3 standard deviations. The signals were subsequently corrected for any DC shift and rectified. The area under the curve (AUC) of the eHFO and lHFO was measured and used for further analysis.

Data were analyzed using a custom MATLAB (MathWorks, Inc., Massachusetts, USA; version R2025b) script.

### 2.7 Statistical analysis

Linear mixed-effects models were used to analyze the CR score, STDT (trained and untrained sides), N20/P25 amplitude, PPD5ms, PPD30ms, HFO single (eHFO_single_, lHFO_single_, and eHFO_single_/lHFO_single_ ratio), HFO ISI5ms (eHFO_ISI5ms_, lHFO_ISI5ms_, and eHFO_ISI5ms_/lHFO_ISI5ms_ ratio), and HFO ISI30ms (eHFO_ISI30ms_, lHFO_ISI30ms_, and eHFO_ISI30ms_/lHFO_ISI30ms_ ratio). A two-factor mixed design analysis was implemented with group (CR, FT) and test (pre, post) as independent variables and the measured values as dependent variables. Fixed effects included group, test, and group × test interaction; participants were treated as random effects to account for within-subject variability in repeated measurements. The Satterthwaite method was used to calculate degrees of freedom. The significance level for main effects and interactions in the linear mixed-effects models was set at *p* < 0.05. Simple main effects were examined when the interactions were significant. Comparisons between the CR and FT groups at the pre- and post-test, and comparisons between pre- and post-test within each group, were performed with Bonferroni correction applied across these four planned pairwise comparisons (significance threshold *p* < 0.0125 = 0.05/4).

To examine the relationships between changes in motor learning–related neurophysiological measures, Pearson's correlation analyses were performed on the pre-to-post changes (Δ values) of the following variables: CR score, STDT (trained side), N20/P25 amplitude, PPD5ms, PPD30ms, HFO single (eHFO_single_, lHFO_single_, and eHFO_single_/lHFO_single_ ratio), HFO ISI5ms (eHFO_ISI5ms_, lHFO_ISI5ms_, and eHFO_ISI5ms_/lHFO_ISI5ms_ ratio), and HFO ISI30ms (eHFO_ISI30ms_, lHFO_ISI30ms_, and eHFO_ISI30ms_/lHFO_ISI30ms_ ratio). Analyses were performed separately for the CR and FT groups. The significance level was set at *p* < 0.05. Correlations among HFO parameters were not examined, as these were not the primary focus of the present analyses. To account for multiple comparisons (55 pairs per group), the Benjamini–Hochberg false discovery rate (FDR) correction was applied to all pairwise correlation p-values within each group separately. Absolute change scores (Δ = post − pre) were used rather than percentage change, as several dependent variables (PPD, HFO AUC) approached zero in some participants, rendering percentage change scores unstable.

All statistical analyses were performed using R (version 4.5.3; R Foundation for Statistical Computing, Vienna, Austria).

## 3 Results

Table 1 presents the estimated marginal means, standard errors, and confidence intervals for each condition.

**Table 1:** Estimated marginal means (M), standard errors (SE), and confidence intervals (CI) for each condition.

		Pre-test			Post-test		
		M (SE)	95% CI		M (SE)	95% CI	
	Group		Lower	Upper		Lower	Upper
CR score	CR	13.40 (0.73)	11.93	14.87	17.80 (0.73)	16.33	19.27
	FT	12.80 (0.73)	11.33	14.27	13.19 (0.73)	11.72	14.66
STDT (trained side) (ms)	CR	37.96 (4.48)	28.95	46.96	29.34 (4.48)	20.34	38.35
	FT	37.54 (4.48)	28.54	46.55	33.42 (4.48)	24.41	42.43
STDT (untrained side) (ms)	CR	31.80 (4.11)	23.51	40.09	34.61 (4.11)	26.32	42.89
	FT	37.37 (4.11)	29.08	45.66	37.19 (4.11)	28.91	45.48
N20/P25 peak-to-peak amplitude (μV)	CR	4.77 (0.55)	3.65	5.88	5.10 (0.55)	3.99	6.22
	FT	4.35 (0.55)	3.23	5.47	4.63 (0.55)	3.52	5.75
PPD5ms (%)	CR	24.41 (6.54)	11.31	37.51	16.86 (6.54)	3.76	29.96
	FT	7.05 (6.54)	−6.05	20.16	11.94 (6.54)	−1.17	25.04
PPD30ms (%)	CR	59.87 (4.12)	51.64	68.09	59.75 (4.12)	51.52	67.97
	FT	73.78 (4.12)	65.56	82.01	63.87 (4.12)	55.65	72.09
eHFO_single_ (AUC) (μV·ms)	CR	0.24 (0.03)	0.18	0.29	0.28 (0.03)	0.22	0.34
	FT	0.21 (0.03)	0.16	0.27	0.27 (0.03)	0.22	0.33
lHFO_single_ (AUC) (μV·ms)	CR	0.24 (0.04)	0.16	0.32	0.29 (0.04)	0.21	0.37
	FT	0.23 (0.04)	0.15	0.31	0.29 (0.04)	0.21	0.37
eHFO_single_/lHFO_single_ ratio	CR	1.32 (0.18)	0.95	1.68	1.29 (0.18)	0.92	1.66
	FT	1.11 (0.18)	0.75	1.48	1.08 (0.18)	0.72	1.45
eHFO_ISI5ms_ (AUC) (μV·ms)	CR	0.20 (0.02)	0.15	0.25	0.25 (0.02)	0.20	0.30
	FT	0.19 (0.02)	0.14	0.24	0.25 (0.02)	0.20	0.30
lHFO_ISI5ms_ (AUC) (μV·ms)	CR	0.27 (0.05)	0.17	0.36	0.39 (0.05)	0.29	0.48
	FT	0.25 (0.05)	0.15	0.34	0.32 (0.05)	0.23	0.42
eHFO_ISI5ms_/lHFO_ISI5ms_ ratio	CR	0.86 (0.13)	0.60	1.12	0.76 (0.13)	0.50	1.03
	FT	1.16 (0.13)	0.90	1.43	0.95 (0.13)	0.69	1.21
eHFO_ISI30ms_ (AUC) (μV·ms)	CR	0.17 (0.02)	0.12	0.22	0.18 (0.02)	0.13	0.23
	FT	0.19 (0.02)	0.14	0.24	0.20 (0.02)	0.15	0.25
lHFO_ISI30ms_ (AUC) (μV·ms)	CR	0.12 (0.03)	0.06	0.17	0.16 (0.03)	0.11	0.21
	FT	0.16 (0.03)	0.11	0.21	0.19 (0.03)	0.14	0.24
eHFO_ISI30ms_/lHFO_ISI30ms_ ratio	CR	1.92 (0.30)	1.31	2.53	1.34 (0.30)	0.74	1.95
	FT	1.73 (0.30)	1.12	2.33	1.35 (0.30)	0.74	1.95

Abbreviations: CR, coin rotation; FT, finger tapping; STDT, somatosensory temporal discrimination threshold; PPD, paired-pulse depression; HFO, high-frequency oscillation; eHFO, early high-frequency oscillation; lHFO, late high-frequency oscillation; ISI, interstimulus interval; AUC, area under the curve.

### 3.1 CR score

**Fig. 1:**
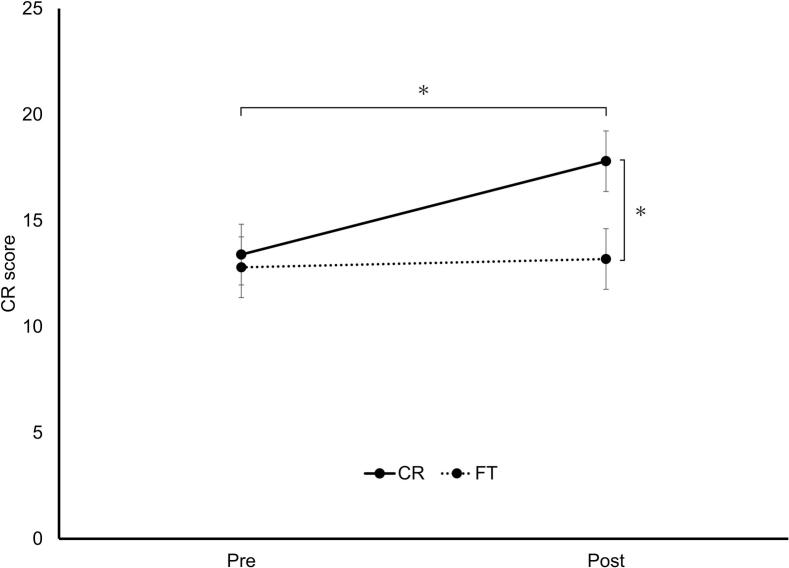
CR performance pre- and post-training. The CR (solid) and FT (dashed) groups are shown. Data represent estimated marginal means ±95% CI. The asterisk above the bracket spanning Pre to Post indicates a significant pre-to-post change in the CR group. The asterisk on the right indicates a significant between-group difference at post-test (pairwise comparisons). *p < 0.05.Abbreviations: CR, coin rotation; CI, confidence interval; FT, finger tapping.

Analysis revealed a significant interaction between group and test (F[1,36] = 43.23, *p* < 0.001, η^2^p = 0.55), a main effect of group (F[1,36] = 7.01, *p* = 0.012, η^2^p = 0.16), and a main effect of test (F[1,36] = 61.72, *p* < 0.001, η^2^p = 0.63). Post-hoc tests showed a significant difference between pre- and post-test results in the CR group (t[36] = 10.20, *p* < 0.001), but not in the FT group, and showed a significant difference between the CR and FT groups at the post-test stage (t[42.85] = 4.48, *p* < 0.001) (Fig. 1).

### 3.2 STDT

**Fig. 2:**
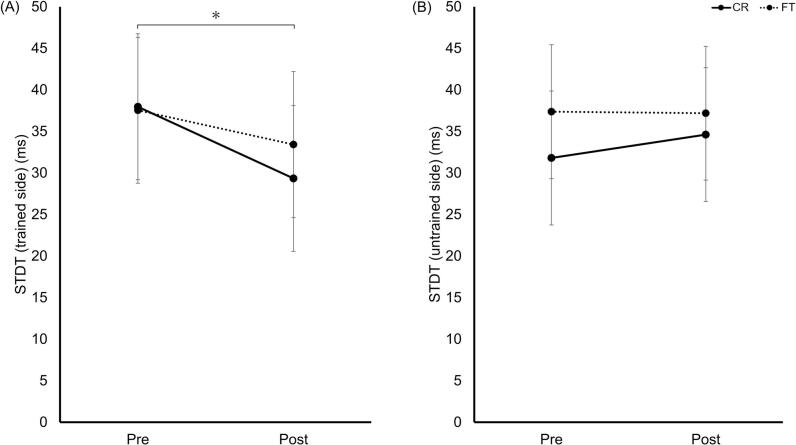
STDT pre- and post-training. (A) STDT trained side; (B) STDT untrained side. The CR (solid) and FT (dashed) groups are shown. Data represent estimated marginal means ±95% CI. The asterisk in panel (A) indicates a significant main effect of test. *p < 0.05. Abbreviations: STDT, somatosensory temporal discrimination threshold; CR, coin rotation; FT, finger tapping; CI, confidence interval.

STDT on the trained side decreased post-training (main effect of test: F[1,36] = 6.23, *p* = 0.017, η^2^p = 0.15). No significant main effect of group or group × test interaction was observed (Fig. 2A).

No significant interactions or main effects were observed for STDT on the untrained side (Fig. 2B).

### 3.3 N 20/P25 amplitude

**Fig. 3:**
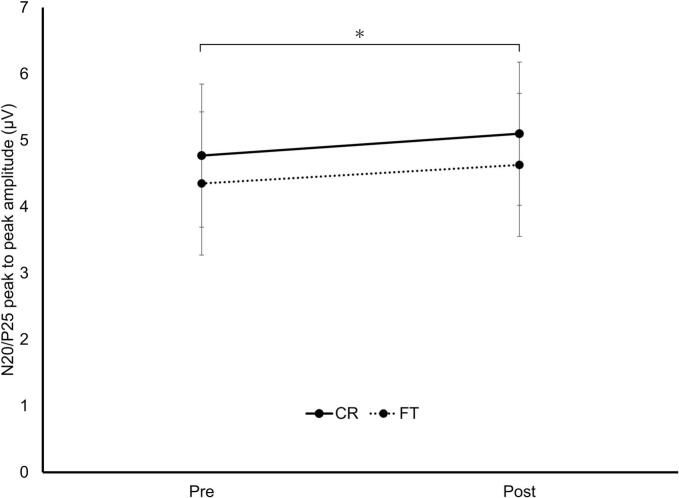
N20/P25 peak-to-peak amplitude pre- and post-training. The CR (solid) and FT (dashed) groups are shown. Data represent estimated marginal means ±95% CI. The asterisk indicates a significant main effect of test. *p < 0.05.Abbreviations: CR, coin rotation; FT, finger tapping; CI, confidence interval.

N20/P25 amplitude significantly increased post-training (main effect of test: F[1,36] = 13.18, *p* < 0.001, η^2^p = 0.27). No significant group effect or group × test interaction was detected (Fig. 3).

### 3.4 PPD

**Fig. 4:**
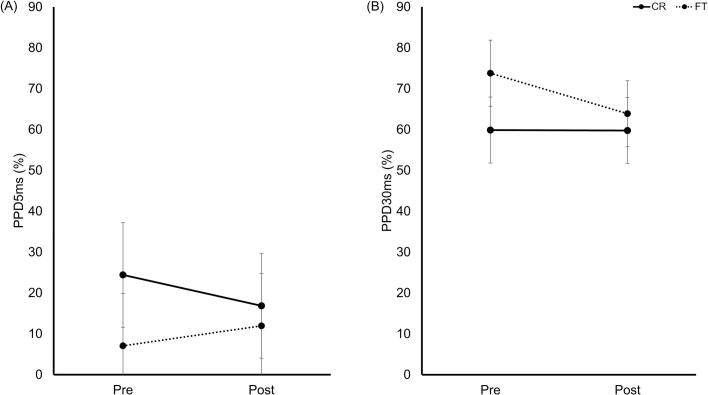
PPD5ms and PPD30ms pre- and post-training. (A) PPD5ms; (B) PPD30ms. The CR (solid) and FT (dashed) groups are shown. Data represent estimated marginal means ±95% CI. Abbreviations: PPD5ms, paired-pulse depression at 5 ms; PPD30ms, paired-pulse depression at 30 ms; CR, coin rotation; FT, finger tapping; CI, confidence interval.

No significant main effects or interactions were observed for PPD5ms and PPD30ms (Fig. 4).

### 3.5 HFO single

**Fig. 5:**
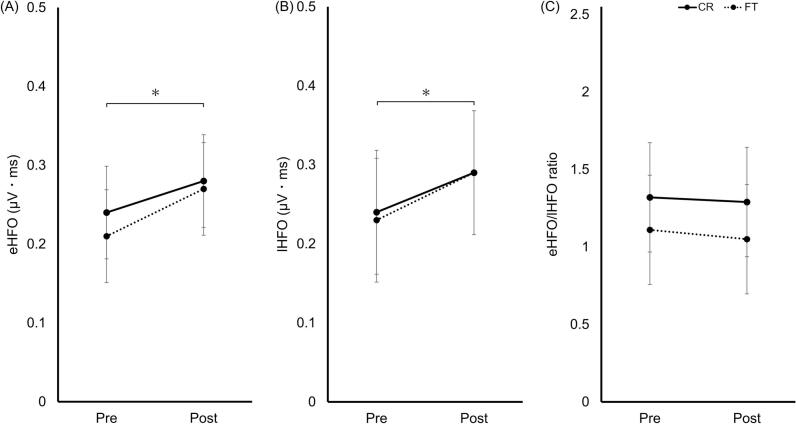
HFO_single_ pre- and post-training. (A) eHFO_single_; (B) lHFO_single_; (C) eHFO_single_/lHFO_single_ ratio. The CR (solid) and FT (dashed) groups are shown. Data represent estimated marginal means ±95% CI. Asterisks in panels (A) and (B) indicate a significant main effect of test. *p < 0.05. Abbreviations: eHFO, early high-frequency oscillation; lHFO, late high-frequency oscillation; HFO, high-frequency oscillation; CR, coin rotation; FT, finger tapping; CI, confidence interval.

eHFO_single_ significantly increased post-training (main effect of test: F[1,36] = 12.63, *p* = 0.001, η^2^p = 0.26). No significant group effect or group × test interaction was detected (Fig. 5A).

lHFO_single_ significantly increased post-training (main effect of test: F[1,36] = 6.20, *p* = 0.018, η^2^p = 0.15). No significant group effect or group × test interaction was detected (Fig. 5B).

No significant main effects or interactions were observed for the eHFO_single_/lHFO_single_ ratio (Fig. 5C).

### 3.6 HFO ISI5ms

**Fig. 6:**
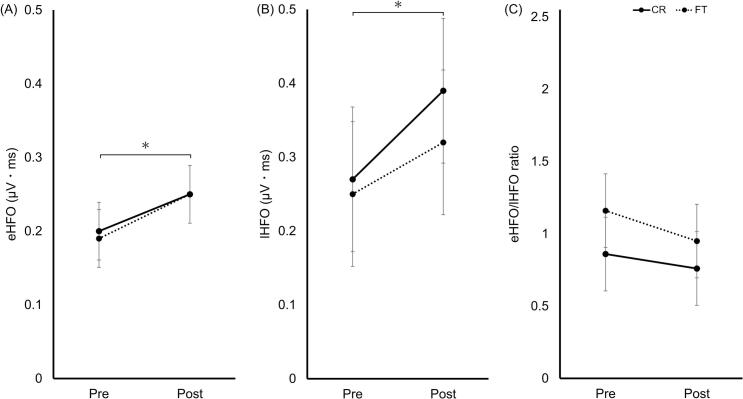
HFO_ISI5ms_ pre- and post-training. (A) eHFO_ISI5ms_; (B) lHFO_ISI5ms_; (C) eHFO_ISI5ms_/lHFO_ISI5ms_ ratio. The CR (solid) and FT (dashed) groups are shown. Data represent estimated marginal means ± 95% CI. Asterisks in panels (A) and (B) indicate a significant main effect of test. *p < 0.05. Abbreviations: eHFO, early high-frequency oscillation; lHFO, late high-frequency oscillation; ISI, interstimulus interval; HFO, high-frequency oscillation; CR, coin rotation; FT, finger tapping; CI, confidence interval.

eHFO_ISI5ms_ significantly increased post-training (main effect of test: F[1,36] = 14.00, *p* < 0.001, η^2^p = 0.28). No significant group effect or group × test interaction was detected (Fig. 6A).

lHFO_ISI5ms_ significantly increased post-training (main effect of test: F[1,36] = 7.78, *p* = 0.008, η^2^p = 0.18). No significant group effect or group × test interaction was detected (Fig. 6B).

No significant main effects or interactions were observed for the eHFO_ISI5ms_/lHFO_ISI5ms_ ratio (Fig. 6C).

### 3.7 HFO ISI30ms

**Fig. 7:**
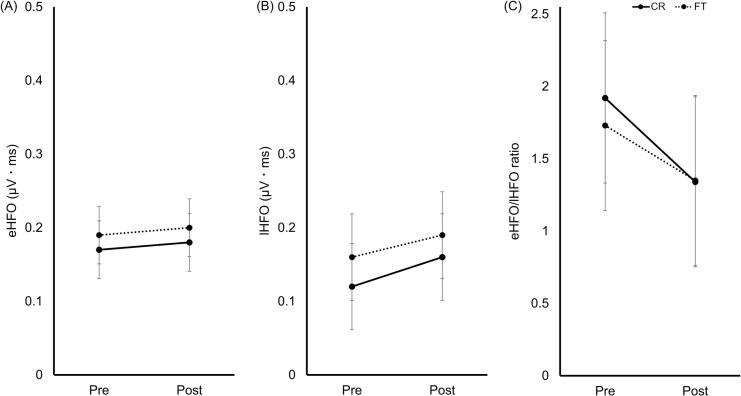
HFO_ISI30ms_ pre- and post-training. (A) eHFO_ISI30ms_; (B) lHFO_ISI30ms_; (C) eHFO_ISI30ms_/lHFO_ISI30ms_ ratio. The CR (solid) and FT (dashed) groups are shown. Data represent estimated marginal means ± 95% CI. Abbreviations: eHFO, early high-frequency oscillation; lHFO, late high-frequency oscillation; ISI, interstimulus interval; HFO, high-frequency oscillation; CR, coin rotation; FT, finger tapping; CI, confidence interval.

No significant main effects or interactions were observed for eHFO_ISI30ms_ (Fig. 7A), lHFO_ISI30ms_ (Fig. 7B), and the eHFO_ISI30ms_/lHFO_ISI30ms_ ratio (Fig. 7C).

### 3.8 Correlation analyses of Pre-to-Post changes (CR Group)

In the CR group, several nominally significant correlations were identified among the Δ values (Table 2). The change in CR score was positively correlated with the change in eHFO_single_ (r = 0.489, *p* = 0.034). The change in STDT (trained side) was positively correlated with the change in lHFO_ISI5ms_ (r = 0.539, *p* = 0.017). The change in N20/P25 amplitude was negatively correlated with the change in eHFO_single_ (r =  − 0.466, *p* = 0.044) and with the change in lHFO_ISI5ms_ (r =  − 0.517, *p* = 0.023). No significant correlations were observed between PPD parameters and any other variables in this group. When Benjamini–Hochberg FDR correction was applied across all 55 pairwise comparisons in the CR group, no correlation remained significant after correction (all q > 0.05; minimum q = 0.607).

**Table 2:** Pearson correlation coefficients and associated p-values for pre-to-post change scores (Δ values) in the coin rotation (CR) group (n = 19). Correlations are presented between CR score, STDT (trained side), N20/P25 peak-to-peak amplitude, PPD5ms, and PPD30ms, and between each of these variables and HFO parameters (single-stimulus, ISI 5 ms, and ISI 30 ms conditions). Correlations among HFO parameters are not shown, as these were not the focus of the present analyses. Values are presented as Pearson's r (p-value). Statistically significant correlations (p < 0.05) are shown in bold and marked with an asterisk (*). Benjamini–Hochberg FDR correction was applied across all 55 pairwise comparisons; no correlation survived correction (minimum q = 0.607).

	CR score		STDT(trained side)		N20/P25 peak-to-peakamplitude		PPD5ms		PPD30ms	
STDT (trained side)	−0.055	(0.823)								
N20/P25 peak-to-peak amplitude	−0.270	(0.264)	−0.346	(0.147)						
PPD5ms	−0.09	(0.714)	0.011	(0.964)	0.027	(0.914)				
PPD30ms	−0.03	(0.902)	0.007	(0.978)	0.191	(0.432)				
eHFO_single_	**0.489**	(0.034)*	0.107	(0.663)	**−0.466**	(0.044)*	−0.044	(0.858)	−0.082	(0.738)
lHFO_single_	0.285	(0.237)	0.012	(0.960)	−0.142	(0.563)	−0.205	(0.400)	−0.336	(0.160)
eHFO_single_/lHFO_single_ ratio	0.124	(0.614)	−0.032	(0.896)	−0.296	(0.219)	0.266	(0.271)	0.342	(0.152)
eHFO_ISI5ms_	0.131	(0.593)	−0.096	(0.697)	−0.159	(0.515)	0.122	(0.619)	0.346	(0.146)
lHFO_ISI5ms_	0.279	(0.248)	**0.539**	(0.017)*	**−0.517**	(0.023)*	−0.072	(0.771)	0.175	(0.474)
eHFO_ISI5ms_/lHFO_ISI5ms_ ratio	−0.273	(0.258)	−0.249	(0.304)	0.178	(0.466)	0.098	(0.689)	−0.162	(0.508)
eHFO_ISI30ms_	−0.046	(0.851)	0.238	(0.326)	−0.276	(0.252)	0.092	(0.707)	0.349	(0.144)
lHFO_ISI30ms_	0.242	(0.318)	0.047	(0.847)	−0.223	(0.359)	0.227	(0.350)	0.154	(0.530)
eHFO_ISI30ms_/lHFO_ISI30ms_ ratio	−0.322	(0.179)	0.315	(0.189)	−0.202	(0.406)	−0.137	(0.576)	0.230	(0.343)

Abbreviations: STDT, somatosensory temporal discrimination threshold; PPD, paired-pulse depression; HFO, high-frequency oscillation; eHFO, early high-frequency oscillation; lHFO, late high-frequency oscillation; ISI, interstimulus interval; CR, coin rotation; FDR, false discovery rate.

### 3.9 Correlation analyses of pre-to-post changes (FT Group)

In the FT group, a different pattern of nominally significant correlations was observed (Table 3). The change in CR score was negatively correlated with the change in PPD5ms (r = −0.521, *p* = 0.022). The change in STDT (trained side) was positively correlated with the change in lHFO_ISI30ms_ (r = 0.509, *p* = 0.026). No significant correlations were observed between HFO parameters and CR score or N20/P25 amplitude in this group. When FDR correction was applied across all 55 pairwise comparisons in the FT group, no correlation remained significant after correction (all q > 0.05; minimum q = 0.716).

**Table 3:** Pearson correlation coefficients and associated p-values for pre-to-post change scores (Δ values) in the finger tapping (FT) group (n = 19). Correlations are presented between CR score, STDT (trained side), N20/P25 peak-to-peak amplitude, PPD5ms, and PPD30ms, and between each of these variables and HFO parameters (single-stimulus, ISI 5 ms, and ISI 30 ms conditions). Correlations among HFO parameters are not shown, as these were not the focus of the present analyses. Values are presented as Pearson's r (p-value). Statistically significant correlations (p < 0.05) are shown in bold and marked with an asterisk (*). Benjamini–Hochberg FDR correction was applied across all 55 pairwise comparisons; no correlation survived correction (minimum q = 0.716).

	CR score		STDT(trained side)		N20/P25 peak-to-peakamplitude		PPD5ms		PPD30ms	
STDT (trained side)	0.158	(0.518)								
N20/P25 peak-to-peak amplitude	−0.050	(0.840)	0.243	(0.317)						
PPD5ms	**−0.521**	(0.022)*	0.166	(0.497)	0.192	(0.431)				
PPD30ms	0.134	(0.584)	0.202	(0.408)	0.059	(0.812)				
eHFO_single_	0.147	(0.547)	0.304	(0.206)	0.195	(0.423)	0.131	(0.593)	0.237	(0.329)
lHFO_single_	0.184	(0.450)	0.333	(0.164)	0.021	(0.932)	0.267	(0.270)	−0.022	(0.928)
eHFO_single_/lHFO_single_ ratio	−0.028	(0.911)	0.029	(0.906)	−0.008	(0.974)	−0.111	(0.650)	0.281	(0.243)
eHFO_ISI5ms_	−0.272	(0.261)	0.190	(0.436)	0.094	(0.701)	0.267	(0.269)	−0.306	(0.202)
lHFO_ISI5ms_	−0.072	(0.770)	0.438	(0.061)	0.193	(0.430)	0.269	(0.265)	−0.366	(0.123)
eHFO_ISI5ms_/lHFO_ISI5ms_ ratio	−0.097	(0.692)	−0.421	(0.073)	−0.235	(0.332)	−0.165	(0.500)	0.282	(0.243)
eHFO_ISI30ms_	−0.305	(0.204)	0.124	(0.612)	0.047	(0.848)	0.062	(0.801)	0.278	(0.249)
lHFO_ISI30ms_	0.019	(0.940)	**0.509**	(0.026)*	0.14	(0.567)	0.145	(0.553)	0.053	(0.830)
eHFO_ISI30ms_/lHFO_ISI30ms_ ratio	−0.035	(0.888)	−0.163	(0.504)	0.183	(0.453)	−0.154	(0.528)	0.048	(0.845)

Abbreviations: STDT, somatosensory temporal discrimination threshold; PPD, paired-pulse depression; HFO, high-frequency oscillation; eHFO, early high-frequency oscillation; lHFO, late high-frequency oscillation; ISI, interstimulus interval; CR, coin rotation; FDR, false discovery rate.

## 4 Discussion

The principal findings are summarized below. A significant main effect of test was observed for trained-side STDT, N20/P25 amplitude, and eHFO and lHFO under the single-stimulus and ISI5ms conditions, with no significant main effect of group or group × test interaction for any of these measures, indicating that pre-to-post changes occurred similarly in both groups following training. In contrast, neither PPD5ms nor PPD30ms showed a significant main effect of test or group × test interaction, nor was a group × test interaction detected for any outcome measure. These results do not support the initial hypothesis that feedback-dependent motor learning would selectively modulate STDT and PPD-indexed S1 intracortical inhibitory processing relative to the FT task. The present data are more compatible with the view that STDT and S1 cortical responses may be commonly modulated by repetitive sensorimotor practice, regardless of task type, rather than reflecting effects specific to feedback-dependent motor learning. The neurophysiological significance of each finding and the interpretive constraints of the present study are discussed below.

### 4.1 Neurophysiological indices: conceptual framework

Before interpreting the present findings, it is useful to clarify the conceptual roles of the neurophysiological indices employed. N20/P25 amplitude and HFO are primarily regarded as indices of cortical excitability, whereas PPD is used as an index of intracortical inhibition. These measures are thought to reflect distinct neural mechanisms and may vary independently of one another (see Section 4.2).

The N20 component is thought to reflect excitatory postsynaptic potentials arising from thalamocortical projections that depolarize the apical dendrites of pyramidal neurons in S1 (Allison et al., 1991). eHFO is considered to primarily reflect high-frequency activity originating from thalamocortical fibers, whereas lHFO is thought to reflect the activity of inhibitory interneuron populations located in Brodmann areas 1 and 3B within S1, particularly those mediating feedforward inhibition (Ozaki and Hashimoto, 2011, Hashimoto et al., 1996, Dubbioso et al., 2022). An alternative view, however, posits that lHFO reflects burst firing of pyramidal tract neurons in BA3B rather than inhibitory interneurons (Baker et al., 2003, Telenczuk et al., 2011, Tomasevic et al., 2022). This interpretive ambiguity warrants caution when inferring specific circuit mechanisms from lHFO amplitude changes. The concurrent increase in eHFO and lHFO therefore raises the possibility of altered S1 response dynamics involving thalamocortical and intracortical components, although this interpretation cannot be directly demonstrated from the present data.

### 4.2 Dissociation between STDT improvement and S1 inhibitory changes

The central finding of the present study is a dissociation between STDT improvement and the absence of change in PPD. A significant reduction in trained-side STDT was observed following the intervention (main effect of test), yet neither PPD5ms nor PPD30ms showed a significant main effect of test or group × test interaction. These results suggest that short-term sensorimotor practice may modulate STDT without necessarily altering S1 inhibitory processing as indexed by PPD.

The neural basis of PPD varies depending on the ISI employed. Inhibition at approximately 5 ms has been proposed to reflect local inhibitory interneuron activity within S1, based on indirect evidence from its associations with STDT and HFO (Rocchi et al., 2016, Rocchi et al., 2017, Antelmi et al., 2017, Erro et al., 2018). In contrast, the neural substrate of inhibition at approximately 30 ms is less well established; contributions from remote structures outside S1, including the dorsal column nuclei (Lüders et al., 1984) and the thalamus (Höffken et al., 2010), have been proposed but not directly demonstrated (see also Section 4.8). Accordingly, the present results suggest that STDT improvement may not require changes in PPD-indexed S1 inhibitory processing at either ISI. Whether subcortical mechanisms contribute to STDT modulation during sensorimotor practice remains an open question.

PPD does not necessarily correspond directly to the absolute amplitude of the N20 component (Gatica Tossi et al., 2013, Höffken et al., 2013), and the two indices are thought to reflect distinct neurophysiological aspects of S1 processing. One possible explanation for the coexistence of increased SEP amplitude and HFO with unchanged PPD is a proportional gain increase: if both the single-stimulus and paired-stimulus responses increased proportionally—consistent with the observed increase in N20/P25 amplitude—the suppression ratio would remain unchanged even as overall cortical response gain increased (Yamazaki et al., 2020). It is also worth noting that PPD is calculated as a ratio between the second and first responses, with the single-stimulus SEP amplitude serving as the denominator; a change in single-stimulus SEP amplitude following an intervention may influence the PPD ratio independently of changes in the paired-pulse response itself (Yamazaki et al., 2020). The simultaneous observation of increased SEP amplitude and unchanged PPD therefore does not represent an internal inconsistency in the present data.

Regarding the increase in N20/P25 amplitude, comparison with prior studies should be interpreted cautiously. Paparella et al. (2020) reported a decrease in SEP amplitude elicited by digital nerve stimulation of the task-involved finger following a ballistic movement task—a direction opposite to that observed in the present study. This discrepancy may be attributable to at least two factors. First, the stimulation site differed: the present study used median nerve stimulation at the wrist, which has a different afferent pathway from digital nerve stimulation targeting cutaneous input directly associated with the task. Selective sensory gating of task-relevant digits would not necessarily preclude an increase in SEP amplitude from a more proximal median nerve stimulus. Second, tasks requiring continuous sensory feedback, such as CR, may promote processing efficiency for incoming sensory information rather than its suppression. These considerations suggest that the direction of SEP amplitude changes following motor learning may be task-specific (Paparella et al., 2020), and the present findings may represent one instance within this broader context.

### 4.3 Intervention modality-specific plasticity patterns

The present neurophysiological pattern—concurrent increases in SEP amplitude and HFO in the absence of PPD change—differs from those reported in other intervention modalities. Rocchi et al. (2016) reported concurrent changes in HFO and PPD at ISI 5 ms following cTBS over S1 in healthy subjects, whereas Rocchi et al. (2017) and Erro et al. (2018) reported concurrent changes in HFO and PPD across multiple ISIs following HF-RSS in healthy subjects and in patients with dystonia. Antelmi et al. (2017) further demonstrated, in a cross-sectional comparison, that reduced HFO and impaired PPD suppression at multiple ISIs were both associated with abnormal STDT in cervical dystonia. Taken together, these findings suggest that reorganization of S1 inhibitory interneuron circuits may be a primary substrate of plasticity in passive sensory stimulation paradigms. By contrast, Yamazaki et al. (2020) reported a reduction in PPD30ms without change in SEP amplitude or STDT following moderate aerobic exercise—a pattern distinct from both of the above and from the present findings. These observations raise the possibility that different intervention modalities may selectively recruit distinct plasticity mechanisms within the somatosensory system, although this tentative classification is based on a limited number of studies and requires prospective verification.

Zhang et al. (2025) reported that SIR—an index of lateral inhibition between adjacent digits in S1, where higher values indicate reduced suppression—increased following both ball-rotation training and motor imagery, and these SIR increases were positively correlated with STDT improvement in a location-specific manner. Our data, by contrast, showed no change in PPD, which indexes temporal paired-pulse suppression rather than spatial surround inhibition. It should be noted, however, that the SIR increase in Zhang et al. (2025) may reflect training-specific reorganization of adjacent digit representations in S1, given that ball-rotation training inherently involves coordinated multi-digit movements, rather than indicating a general susceptibility of spatial inhibitory circuits to sensorimotor practice; direct comparison between the two studies is therefore limited.

Taken together, the absence of change in PPD5ms and PPD30ms combined with the reduction in STDT—and the consistent finding of Paparella et al. (2020), who reported no change in paired-pulse SEP suppression across two different motor learning paradigms—suggest that PPD-indexed inhibition may be relatively resistant to transient voluntary sensorimotor practice, in contrast to the changes induced by passive sensory stimulation paradigms such as HF-RSS (Rocchi et al., 2017, Erro et al., 2018). The present data further raise the possibility that STDT modulation during sensorimotor practice may reflect a generalized increase in response gain across sensory processing circuits, rather than a selective change in the inhibitory ratio indexed by PPD within local S1 circuits or remote inhibitory loops. This interpretation remains speculative, and more targeted interventional studies will be required to obtain direct supporting evidence.

### 4.4 Absence of between-group differences and its implications

Contrary to our hypothesis, no significant group × test interactions were observed for any outcome measure. The FT task was selected as an active comparison task to match motor activity and training duration while imposing lower intended feedback demands than CR. However, both tasks may recruit partially overlapping sensorimotor networks, potentially including cortical and subcortical components implicated in STDT regulation (Lee et al., 2018, Ordás and Alonso-Frech, 2024, Witt et al., 2008, Turesky et al., 2018). Even without external synchronization, FT inherently requires temporal motor control (Avanzino et al., 2016), potentially activating cortical and subcortical gating circuits that substantially overlap with those engaged by CR.

At least two explanations for the absence of between-group differences warrant consideration. First, both tasks may have recruited a shared temporal processing network whose generalized activation produced comparable changes in both groups. Second, the training duration—10 sessions of 2 min each—may have been insufficient to unmask task-specific differences that might emerge with more prolonged or intensive practice. Critically, the absence of a passive control group precludes definitive attribution of the observed changes to sensorimotor practice per se (see Section 4.8). These considerations suggest that it would be premature to conclude that feedback-dependent motor learning selectively modulates STDT or S1 plasticity relative to an active comparison task.

### 4.5 Isi-dependency and local circuit involvement

The selective increase in HFO under single-stimulus and ISI5ms conditions, but not ISI30ms, is broadly consistent with the mechanistic framework proposed by Rocchi et al. (2016), in which feedforward inhibitory circuits indexed by l-HFO and PPD at ISI 5 ms are functionally linked, whereas PPD at longer ISIs is thought to reflect distinct, non-cortical inhibitory mechanisms. Within this framework, feedforward inhibition was specifically proposed to contribute to two separable functions: sharpening the temporal profile of cortical excitation (relevant to STDT) and attenuating responses to subsequent inputs at ultra-short latencies (relevant to PPD5ms). The present finding—that lHFO increased under both single-stimulus and ISI5ms conditions while PPD5ms remained unchanged—raises the possibility that these two functions may be dissociable following sensorimotor practice, with feedforward inhibitory activity potentially enhanced—assuming that lHFO increases reflect inhibitory interneuron activity—yet without a corresponding change in paired-pulse suppression. However, if lHFO instead reflects burst firing of pyramidal neurons (Baker et al., 2003, Telenczuk et al., 2011), this interpretation would not hold. Regardless, the absence of robust individual-level associations between HFO changes and STDT improvement (see Section 4.6) precludes stronger mechanistic inference.

### 4.6 Exploratory correlation analyses

Although some significant correlations were observed, these were limited, exploratory, and not systematic across groups or measures, consistent with Paparella et al. (2020), who showed that changes in cortical excitability did not clearly co-vary with motor performance across learning tasks (see also Section 4.3). This lack of systematic co-variation suggests that S1 excitability changes may not directly reflect individual differences in motor skill acquisition in the context of short-term sensorimotor practice examined here, but may instead reflect a more general response to repetitive sensorimotor experience, though this interpretation requires confirmation in studies with passive control conditions. The exploratory correlation analyses reported below should be interpreted in this light.

No correlation survived Benjamini–Hochberg FDR correction in either group; accordingly, all findings should be considered hypothesis-generating and require replication. In the CR group, three of the four nominally significant correlations—ΔSTDT × ΔlHFO_ISI5ms_, ΔN20/P25 × ΔlHFO_ISI5ms_, and ΔN20/P25 × ΔeHFO_single_—became negligible upon exclusion of a single participant with atypically large changes (r = 0.048, −0.10, and −0.297, respectively; all *p* > 0.20), indicating no systematic relationships among these variables. Importantly, the main effects of test observed in the primary linear mixed-effects model analyses were unaffected by the exclusion of this participant (see Section 4.8), suggesting that the primary findings are robust, whereas the exploratory correlational results are particularly sensitive to individual data points. The one exception was the association between ΔCR score and ΔeHFO_single_, which remained after exclusion (r = 0.526, *p* = 0.025) and may warrant further investigation. In the FT group, nominal correlations between ΔCR score and ΔPPD5ms (r =  − 0.521) and between ΔSTDT and ΔlHFO_ISI30ms_ (r = 0.509) were observed but lacked counterparts in the CR group and should not be interpreted as mechanistic evidence.

### 4.7 Magnitude of STDT changes and comparison with prior studies

For reference, prior studies in healthy individuals have reported STDT changes of approximately +12 ms (+16%) following continuous theta burst stimulation (cTBS) to S1 and approximately −12.7 ms (−16%) following intermittent TBS to S1 (Conte et al., 2012), approximately +25.3 ms (+50%) immediately following cTBS (Rocchi et al., 2016), and approximately −19.0 ms (−22%) following HF-RSS (Rocchi et al., 2017). In motor learning paradigms, reductions of approximately −7.2 ms (−17%) following CR (Yoshida et al., 2020) and approximately −20.3 ms (−32%) following ball rotation (Zhang et al., 2025) have been reported.

In the present study, STDT changed from 37.96 ms to 29.34 ms in the CR group (−8.6 ms; −23%) and from 37.54 ms to 33.42 ms in the FT group (−4.1 ms; −11%). The magnitude of change in the CR group is broadly comparable to that reported in previous motor learning studies. The smaller absolute and relative change in the FT group, although not statistically different from the CR group, may simply reflect numerical variation in the absence of a statistically reliable between-group difference; no meaningful interpretation should be drawn from this numerical difference alone. It should also be noted that the participants in the present study showed baseline STDT values at the lower end of the reported normative range (approximately 27–78 ms; Lee et al., 2018), which may have constrained the potential for improvement. Additionally, differences in STDT measurement methodology across studies—the present study adopted the method described by Lacruz et al. (1991), which is associated with baseline values in the range of 30–40 ms—may affect the comparability of absolute change scores.

### 4.8 Limitations

This study has some methodological limitations that should be acknowledged. First, the absence of a passive control group precludes definitive attribution of the observed changes specifically to sensorimotor practice, as contributions from non-specific factors such as the passage of time, repeated measurement, or fatigue cannot be excluded. Second, no direct measures of subcortical activity were obtained, and any involvement of subcortical mechanisms in the observed changes can only be inferred indirectly. Third, the ISI settings used in the present study (5 ms and 30 ms) capture only a limited portion of the inhibitory profile of S1; inclusion of additional ISIs—particularly 20 and 40 ms, which have been employed to probe thalamocortical contributions to paired-pulse SEP suppression (Rocchi et al., 2016, Rocchi et al., 2017, Antelmi et al., 2017, Erro et al., 2018, Dubbioso et al., 2022)—would allow more direct assessment of whether subcortical circuits—including inhibitory interneurons within the dorsal column nuclei (Lüders et al., 1984) and the thalamus, particularly the ventral posterolateral nucleus (Lueders et al., 1983, Höffken et al., 2010)—are engaged during sensorimotor practice. Fourth, the sample size of n = 19 per group limits the statistical power of the correlation analyses, and the exploratory correlational findings reported in this study require replication in larger, independent cohorts before any mechanistic interpretations can be drawn. Although the main effects of test were robust to the exclusion of one participant with atypically large changes, the exploratory correlation analyses were sensitive to this individual, underscoring the need for caution in interpreting these findings (see Section 4.6).

## 5 Conclusions

A main effect of test was observed for trained-side STDT and S1 cortical response measures following short-term sensorimotor practice, without detectable changes in PPD-indexed intracortical inhibition. Contrary to the initial hypothesis, no between-group differences were observed for any outcome measure, suggesting that the observed neurophysiological changes did not show evidence of specificity to feedback-dependent motor learning and may instead reflect a common response to repetitive sensorimotor experience. These findings should be interpreted with caution given the brief training duration, the absence of a passive control group, and the possibility that both tasks engaged substantially overlapping neural networks. Future studies incorporating passive control conditions, extended training protocols, and direct measures of subcortical activity will be needed to clarify the neural mechanisms underlying STDT modulation during sensorimotor practice.

## Funding

This study was supported by JSPS KAKENHI (grant number 23K16614).

## Declaration of Competing Interest

The authors declare that they have no known competing financial interests or personal relationships that could have appeared to influence the work reported in this paper.
